# Assessment of long COVID-19 symptoms and functional status: insights from a cross-sectional study

**DOI:** 10.3389/fmed.2025.1715786

**Published:** 2025-12-17

**Authors:** Nicolás Escrivá, Laura Moreno-Galarraga, Elena Barado, María Gabriela Torres, Alejandro Fernández-Montero

**Affiliations:** 1 Department of Occupational Medicine, Hospital Universitario de Navarra, Navarra, Spain; 2 Instituto de Salud Pública y Laboral de Navarra, Navarra, Spain; 3 Department of Pediatrics, Hospital Universitario de Navarra (HUN), Science Faculty, Medicine, Universidad Pública de Navarra (UPNA), Pamplona, Spain; 4 Navarra Institute for Health Research (IdiSNA), Pamplona, Spain; 5 Department of Occupational Medicine, University of Navarra, Navarra, Spain

**Keywords:** COVID-19, long covid symptoms, post-COVID functional status scale, fatigue, functional impairment

## Abstract

This cross-sectional study examines the functional limitations of Long COVID (LC) in a clinically confirmed cohort (*n* = 220). We collected sociodemographic, clinical, and lifestyle data via a structured electronic form and assessed daily limitations using the Post-COVID-19 Functional Status (PCFS) scale. Linear models were used to evaluate the association between symptom burden and functional limitations and to identify symptom-specific predictors of impairment. Participants had a mean age of 44.8 years, and 80.5% were women. A dose–response pattern linked higher symptom counts with worse PCFS grades in the multivariable-adjusted model (*β* = 0.17; 95% CI 0.10–0.25; *p* < 0.001). In hierarchical models, fatigue, dizziness, and memory loss were independent predictors of greater functional limitations (crude *β*: fatigue 1.56; 95% CI 1.22–1.90; dizziness 1.08; 95% CI 0.81–1.34; and memory loss 1.26; 95% CI 0.97–1.55), cumulatively explaining 51.3% of the variance in functional limitations. In contrast, other common LC symptoms did not retain independent associations after adjustment. These findings highlight the value of simple symptom counts and targeted symptom profiles for risk stratification in primary care and occupational health and for planning rehabilitation and work ability assessment. Prospective studies should validate these indicators over time and explore the mechanisms linking neurocognitive and fatigue phenotypes with persistent disability.

## 1 Introduction

Global understanding of COVID-19, the disease caused by SARS-CoV-2, continues to expand. Beyond the initial impact of this illness, which has resulted in approximately 15 million deaths (1), new public health challenges have emerged. One of the most significant challenges is the persistence of symptoms in a substantial percentage of survivors, currently estimated to affect approximately 6.2% (ranging from 2.4 to 13.3%) (2–4).

The Centers for Disease Control and Prevention (CDC) and the World Health Organization (WHO) define Long COVID (LC) as a broad range of physical or psychological symptoms that begin during or after acute infection, persist for at least 2 months after the acute phase (3 months from symptom onset), and significantly affect daily functioning without being explained by other diagnoses (5, 6). Some authors distinguish between “long-COVID” (symptoms lasting from 4 weeks to 3 months) and “post-COVID” (symptoms persisting beyond 3 months). To avoid terminological ambiguity, in this manuscript, we use the term “Long COVID” throughout to refer to persistent symptoms lasting ≥3 months.

Multiple studies have shown that LC is associated with significant functional limitations, prompting increasing scientific interest in objectively measuring its impact. Common symptoms linked to functional limitations include fatigue, dyspnea, cognitive dysfunction, musculoskeletal pain, and sleep disorders (7–13). These symptoms often reduce patients’ capacity to perform occupational, personal, and social activities, severely affecting both physical and mental well-being (7, 9, 10).

Given the diversity of symptoms observed in LC, several functional assessment tools have been developed to objectively evaluate their impacts. Among these, the Post-COVID-19 Functional Status Scale (PCFS) has gained wide acceptance. This scale categorizes functional limitations from no limitations to severe restrictions in autonomy and has been validated for use in both hospitalized and non-hospitalized populations (7, 11, 13). Other well-established tools, such as the SF-36 and EQ-5D-5L, are frequently used to assess health-related quality of life and functional limitations, covering the physical, psychological, and social domains (10, 12). Additionally, instruments such as the Fatigue Severity Scale (FSS) and the Mental Fatigue Scale (MFS) are employed to measure the severity of fatigue (7, 9).

A key challenge in clinical practice is the difficulty in predicting symptoms that result in greater functional limitations. Currently, there are no specific “alarm symptoms” that reliably help us identify functional decline (4, 14), complicating early identification of high-risk individuals and delaying appropriate interventions.

In this cross-sectional study, we aimed to assess the prevalence and characteristics of LC symptoms and to evaluate the functional limitations of affected individuals using the PCFS Scale. We also aim to identify which persistent symptoms are most strongly associated with poorer long-term functional outcomes in patients with Long COVID.

Our findings are intended to assist healthcare professionals in identifying patients at a higher risk of functional limitations, supporting decisions regarding rehabilitation, follow-up care, and optimal resource allocation.

## 2 Methodology

### 2.1 Study definition

This cross-sectional observational study, nested in a retrospective COVID-19 cohort, was designed to assess the prevalence and characteristics of Long COVID (LC) symptoms and to evaluate their association with functional status. This study aimed to analyze how symptom burden relates to functional limitations and which persistent symptoms are most strongly associated with poorer long-term functional outcomes in patients with Long COVID.

Data collection was conducted using a structured electronic form that included self-reported values, including sociodemographic, clinical, and lifestyle variables.

The study design and reporting followed the Strengthening the Reporting of Observational Studies in Epidemiology (STROBE) guidelines to ensure transparency and scientific rigor (Supplementary Table 1). Confidentiality and data protection were managed in a REDCap (Research Electronic Data Capture) database hosted on a secure institutional server, a web-based application for research data collection and management.

### 2.2 Population definition

Participants, aged over 5 years, were recruited from two main sources: the general outpatient population and members of the Long COVID support associations. Eligibility was contingent upon documented evidence of prior acute SARS-CoV-2 infection. Specifically, inclusion required laboratory confirmation using either PCR or antigen testing. Therefore, participants without a positive diagnostic test and those relying solely on clinical suspicion were excluded.

**Figure 1:**
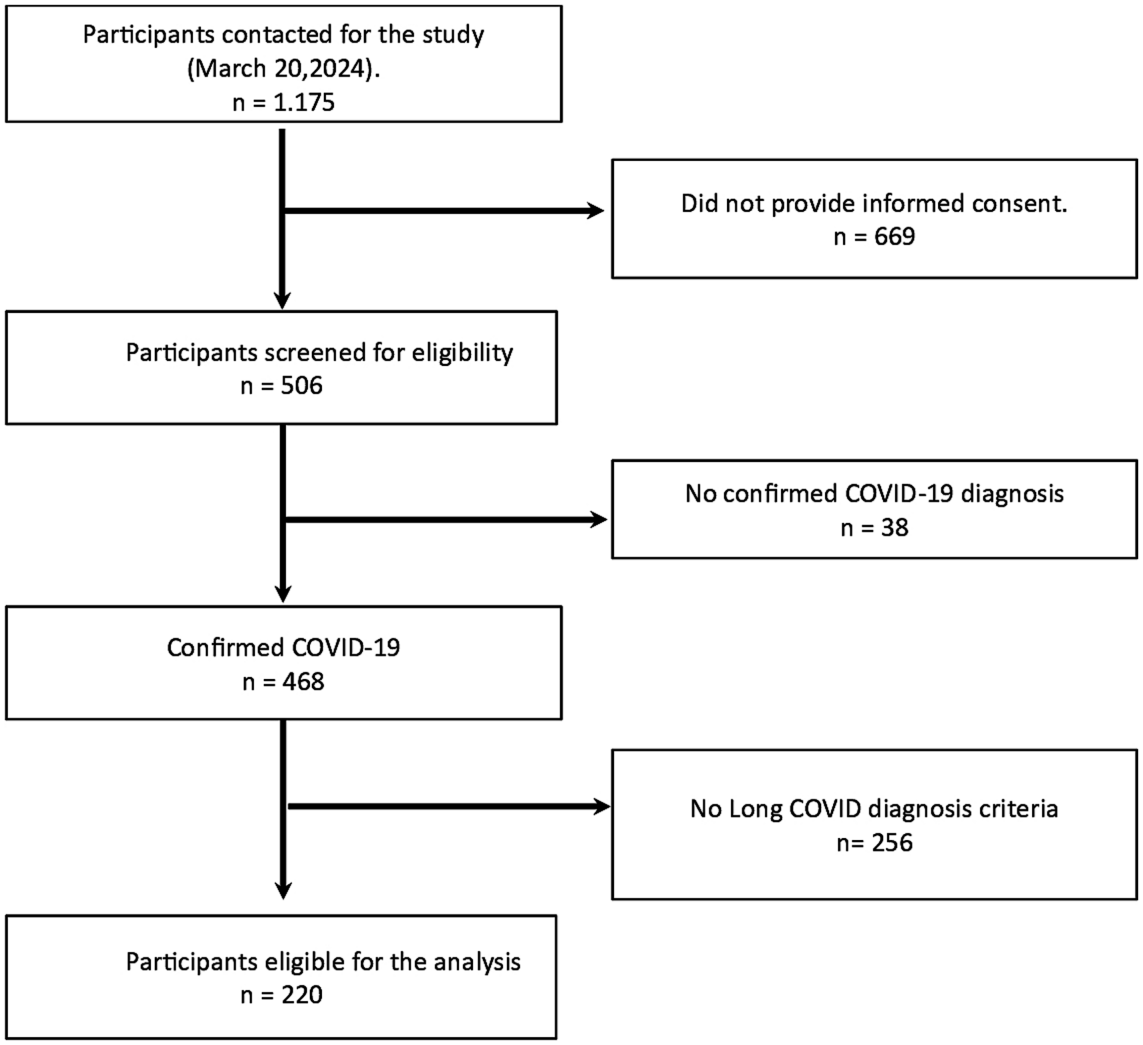
Study flowchart and eligibility criteria. Flow of participants with inclusion/exclusion reasons and the final sample (*n* = 220).

As depicted in Figure 1, individuals who did not provide informed consent, those lacking a confirmed COVID-19 diagnosis, or whose responses were insufficient to determine the presence or absence of Long COVID were excluded from the final analysis. After applying these criteria, 220 participants were included in the study.

### 2.3 Variables

The independent variables of this study were Long COVID symptoms and the total number of reported Long COVID symptoms. This information was collected using a multiple-choice table in which participants could select symptoms from a predefined list of the symptoms most frequently associated with LC in published studies on the subject (3, 15), which included persistent cough, fatigue, shortness of breath, dizziness, concentration difficulties or “brain fog,” memory loss, sleep disturbances, anxiety, depression, alterations in taste or smell, digestive problems, muscle or joint pain, menstrual irregularities, and cardiac problems. For each selected symptom, the participants were asked to specify its duration. Additionally, an open-ended option allowed participants to report any other symptoms that were not included in the list (Supplementary Table 2).

The primary outcome of this study was the functional limitations of participants evaluated using the Post-COVID-19 Functional Status (PCFS) Scale. The PCFS is a validated ordinal scale designed to quantify the functional impact of persistent symptoms following COVID-19 infection (16, 17). The scale classifies functional limitations into five grades based on the patient’s self-perceived ability to perform activities they engaged in prior to SARS-CoV-2 infection: Grade 0: No functional limitations; Grade 1: Negligible functional limitations; Grade 2: Slight functional limitations; Grade 3: Moderate functional limitations; Grade 4: Severe functional limitations (18). For symptom-specific analysis, a dichotomous variable was created based on the PCFS scale, classifying individuals as having severe functional limitations (PCFS levels 3 or 4) or no severe impairment (PCFS levels 0 to 2).

Variables related to status prior to acute SARS-CoV-2 infection were also collected. Sociodemographic variables included age, sex, country of origin, ethnicity, educational level, and employment status as a healthcare worker. Participants were also asked to report their medical history prior to the acute phase of COVID-19, including any preexisting conditions. For analytical purposes, previous disease was operationalized as a binary variable (yes/no) of any condition present before SARS-CoV-2 infection, including but not limited to cancer, chronic pulmonary diseases (asthma, COPD, pulmonary fibrosis, pulmonary hypertension), cardiovascular disorders (cardiomyopathy, congenital heart disease, heart failure, coronary artery disease, stroke), metabolic conditions (diabetes, obesity, liver, or renal disease), and neuropsychiatric conditions such as depression and other illnesses.

In terms of lifestyle habits before infection, the questionnaire covered tobacco use (categorized as current, former, or never smoker), alcohol intake (in grams per day), physical activity and sedentary behavior (in MET/h/week), and dietary quality (assessed using the MEDAS score) (19), average hours of sleep, and body mass index (BMI). Additionally, information regarding vaccination status at the time of infection was collected, including the number of doses received, vaccine type, and administration date. The date of the initial COVID-19 diagnosis and any documented reinfections were also recorded. Participants were also asked to specify after which SARS-CoV-2 infection their persistent Long COVID symptoms appeared (after the first, second, third, or later COVID infection). Based on the reported infection date, each case was classified according to the dominant SARS-CoV-2 variant circulating in Spain during that period, based on prior literature (20), defining four pandemic periods: first wave (March 8–May 30, 2020), pre-vaccination (June 1–December 31, 2020), Delta predominance (January 1–December 15, 2021), and Omicron emergence (December 16, 2021–January 31, 2022). Participants who were unsure or did not provide an answer to this question were conservatively classified as having developed Long COVID symptoms after their first SARS-CoV-2 infection.

### 2.4 Statistical analysis

To describe the baseline characteristics of the sample, categorical variables were reported as percentages, whereas continuous variables were summarized using means and standard deviations (SD).

To explore the association between the number of Long COVID symptoms and functional limitations, we conducted linear regression analyses, including unadjusted, age- and sex-adjusted, and multivariable-adjusted models, based on previous studies (13, 21) and the variables that modify the effect between the independent variable and the outcome. The main independent variable (number of symptoms) and the outcome variable (PCFS score) had no missing values. Consequently, all analyses were performed on the same analytic sample (*N* = 220), including imputed adjustment variables where applicable.

The multivariable-adjusted model included the following covariates: age, sex, preexisting disease, body mass index (BMI), smoking status, alcohol intake, physical activity, sedentary hours per day, sleep duration, adherence to the Mediterranean diet (MEDAS score), and vaccination status for both influenza and COVID-19. The results are presented as beta coefficients with corresponding 95% confidence intervals (CIs) to reflect the precision of the estimates.

To assess the individual contribution of each symptom to variability in the PCFS, a linear regression (crude) model was constructed. Beta coefficients were used to estimate the strength of the association for each symptom, and the cumulative R^2^ indicated the proportion of explained variability.

For missing values in the adjustment variables, multiple imputation by chained equations (MICE) was applied to minimize bias due to incomplete data and ensure compliance with Rubin’s rules. Variables with missing values included age, adherence to the Mediterranean diet, preexisting disease, hours of sleep, and sedentary behavior. Imputation models were specified according to the nature of each variable: logistic regression for binary variables, ordinal logistic regression for ordinal variables, and predictive mean matching (five nearest neighbors) for continuous variables. Twenty imputed datasets were generated, and estimates were combined using Rubin’s rules. Overall, the proportion of patients with missing data was low (<10%).

All *p*-values were calculated using two-tailed tests, with statistical significance defined as *p* < 0.05. Data were processed and analyzed using Stata software, version 17.0.

The study received approval from the Ethics Committee of the University of Navarra (Ref. 2023.042), adheres to the 2023 Medical Data Protection Law, and complies with the principles outlined in the Declaration of Helsinki. All participants provided written informed consent before participation.

To enhance the clarity of the manuscript, ChatGPT (OpenAI) was used during the drafting and translation processes. The final version was thoroughly reviewed and edited by the authors, who assume full responsibility for its content.

## 3 Results

A total of 220 participants with confirmed SARS-CoV-2 infection and persistent symptoms consistent with Long COVID were eligible for analysis. The mean age of the cohort was 44.8 years (SD = 11.0), and 80.5% were women. Regarding functional status, as assessed by the Post-COVID-19 Functional Status (PCFS) scale, 11.8% of participants had grade 1 limitation (negligible), 9.1% reported grade 2 limitation (slight), 21.8% had grade 3 limitation (moderate), and 49.6% had grade 4 limitation (severe) in their daily activities.

**Figure 2:**
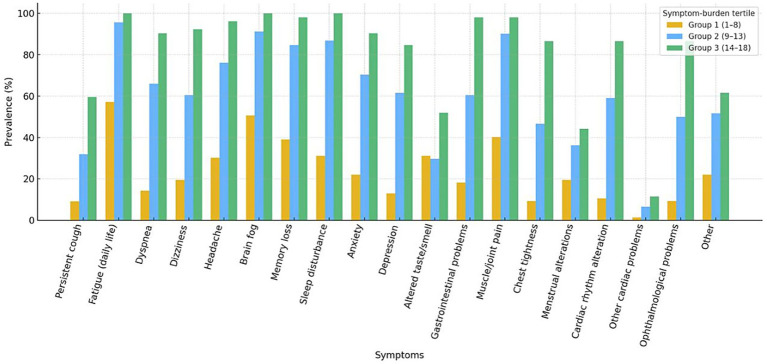
Distribution of key Long COVID symptoms across symptom burden tertiles.

Table 1 presents the baseline characteristics stratified into tertiles according to the number of reported Long COVID symptoms: Group 1 (1–8 symptoms), Group 2 (9–13 symptoms), and Group 3 (14–18 symptoms). To illustrate symptom patterns by symptom burden tertiles, Figure 2 depicts the prevalence of Long COVID symptoms across groups. Fatigue was reported by approximately 95% of the participants in Group 3 compared to 55% in Group 1, with notable differences also observed for persistent cough (~60% vs. ~5%) and dyspnea or dizziness (~90% vs. ~5%). Cognitive symptoms, such as brain fog and memory loss, were also more frequent in Groups 2 and 3.

**Table 1:** Baseline characteristics of participants stratified by tertiles of Long COVID symptoms count.

Variable	All participants (*n* = 220)	Group 1 (1–8 symptoms)	Group 2 (9–13 symptoms)	Group 3 (14–18 symptoms)	*p*-value
Number, *n* (%)	220 (100)	77 (34.4)	91 (41.74)	52 (23.85)	
Age (years), mean (SD)	44.8 (11.0)	43.73 (13.93)	44.92 (9.76)	45.95 (7.99)	*p* = 0.64
Sex, woman, %	80.5 (177/220)	77.92% (60/77)	82.42% (75/91)	78.85% (41/52)	*p* = 0.74
Marital status, married or in a domestic partnership, %	56.8	57.14% (44/77)	59.34% (54/91)	51.92% (27/52)	*p* = 0.69
Race, %					*p* = 0.51
Caucasian	86.9	85.71% (66/77)	87.91% (80/91)	86.54% (45/52)	
Latin-American	4.4	3.9% (3/77)	2.2% (2/91)	7.69% (4/52)	
Other	8.7	10.38% (8/77)	9.89% (9/91)	5.77% (3/52)	
University studies, %	72.2	67.53% (52/77)	75.82% (69/91)	73.08% (38/52)	*p* = 0.55
Employment status, %					*p* = 0.16
Worker	83.1	75.32	87.91	86.54	
Unemployed	9.6	15.58	8.79	5.77	
COVID-19 area worker, %	9.1	5.19% (4/77)	12.09% (11/91)	9.62% (5/52)	*p* = 0.32
Health care worker, %	22.3	25.97% (20/77)	23.08% (21/91)	17.31% (9/52)	*p* = 0.4
Severe COVID-19 symptoms, %	27.3	11.69% (9/77)	36.26% (33/91)	38.46% (20/52)	*p* < 0.001
Infection after which Long COVID symptoms first appeared, %					*p* = 0.02
First	45.9	41.6% (32/77)	45.1% (41/91)	53.8% (28/52)	
Second	13.2	6.5% (5/77)	14.3% (13/91)	21.2% (11/52)	
Third or later	40.9	51.9% (40/77)	40.7% (37/91)	25.0% (13/52)	
Infection period, %					*p* = 0.09
First wave (March–May 2020)	33.2	28.57% (22/77)	31.87% (29/91)	42.31% (22/52)	
Pre-vaccination (June–December 2020)	21.6	15.58% (12/77)	27.47% (25/91)	21.15% (11/52)	
Delta predominance (January–December 15, 2021)	31.9	35.06% (27/77)	29.67% (27/91)	30.77% (16/52)	
Omicron emergence (December 16, 2021–January 31, 2022)	12.3	20.78% (16/77)	10.99% (10/91)	5.77% (3/52)	
BMI, mean (SD)	24.7 (5.1)	23.3 (3.7)	25.4 (5.9)	26.1 (4.4)	*p* = 0.006
Previous disease	46.4	35.06% (27/77)	48.35% (44/91)	58.33% (28/52)	*p* = 0.03
Tobacco use, % ^(a)^					*p* = 0.26
Smoker	12.7	10.39% (8/77)	8.79% (8/91)	19.23% (10/52)	
Former smoker	6.8	6.49% (5/77)	8.79% (8/91)	3.85% (2/52)	
Non-smoker	73.6	74.03% (57/77)	78.02% (71/91)	69.23% (36/52)	
Previous influenza vaccination, %	42.3	49.35% (38/77)	40.66% (37/91)	38.46% (20/52)	*p* = 0.38
Previous COVID-19 vaccination, %	27.3	37.66% (29/77)	26.37% (24/91)	19.23% (10/52)	*p* = 0.06
Alcohol intake ^(β)^, mean (SD)	2.4 (3.9)	2.52 (3.22)	2.27 (4.41)	2.45 (4.16)	*p* = 0.92
Sleep hours per day, mean (SD)	7.6 (1.1)	7.63 (1.07)	7.66 (1.09)	7.46 (1.21)	*p* = 0.62
Sitting hours per day, mean (SD)	6.7 (3.3)	6.95 (3.15)	5.77 (3.07)	7.33 (3.62)	*p* < 0.001
Physical activity ^(c)^, mean (SD)	17.8 (20.3)	14.39 (15.78)	19.69 (21.38)	18.22 (23.39)	*p* = 0.26
Adherence to the Mediterranean diet ^(δ)^	7.2	6.99	7.14	7.46	*p* = 0.51

Values have been calculated based on the number of valid responses. N, number; BMI, body mass index; SD, standard deviation. (a) Percentages are calculated using group totals; sums may be <100% due to missing values. (β) Expressed in grams/day; (c) Expressed in MET-hours/week; (δ) MEDAS Score: Mediterranean Diet Adherence Screener (19).

The mean age and proportion of women were similar across the three groups. No statistically significant differences were observed in marital status, race/ethnicity, educational level, or employment status.

A higher proportion of participants in Groups 2 and 3 had experienced severe acute COVID-19, defined as the presence of pneumonia and/or hospital admission to the ward or intensive care unit (36.26 and 38.46%, respectively), compared to Group 1 (11.69%), with a significant association (*p* < 0.001). Similarly, the prevalence of preexisting medical conditions increased across the groups (*p* = 0.03).

Regarding the infection after which Long COVID symptoms first appeared, the majority of participants reported onset after the first infection (*n* = 101; 45.9%), while 29 (13.2%) and 90 (40.9%) of them developed persistent symptoms after the second or a subsequent infection, respectively. Based on the reported infection dates, the participants were categorized according to the dominant SARS-CoV-2 variant circulating in Spain during that period. Infections were distributed across the first wave, pre-vaccination, Delta, and Omicron phases, with no significant differences across symptom tertiles (*p* = 0.09). However, participants in the highest symptom burden tertile (Group 3) were less represented during the Omicron period, suggesting a possible downward trend in symptom burden in later waves of infection.

Mean BMI increased progressively across tertiles of symptom burden (23.3 ± 3.7, 25.4 ± 5.9, and 26.1 ± 4.4 kg/m^2^ for Groups 1–3, respectively, *p* = 0.006). Participants with higher functional impairment (Groups 2 and 3) had higher BMI values than those in Group 1.

No significant differences were observed across the groups in smoking status, influenza or COVID-19 vaccination, alcohol consumption, sleep duration, or physical activity; adherence to the Mediterranean diet was also comparable across the groups.

**Figure 3:**
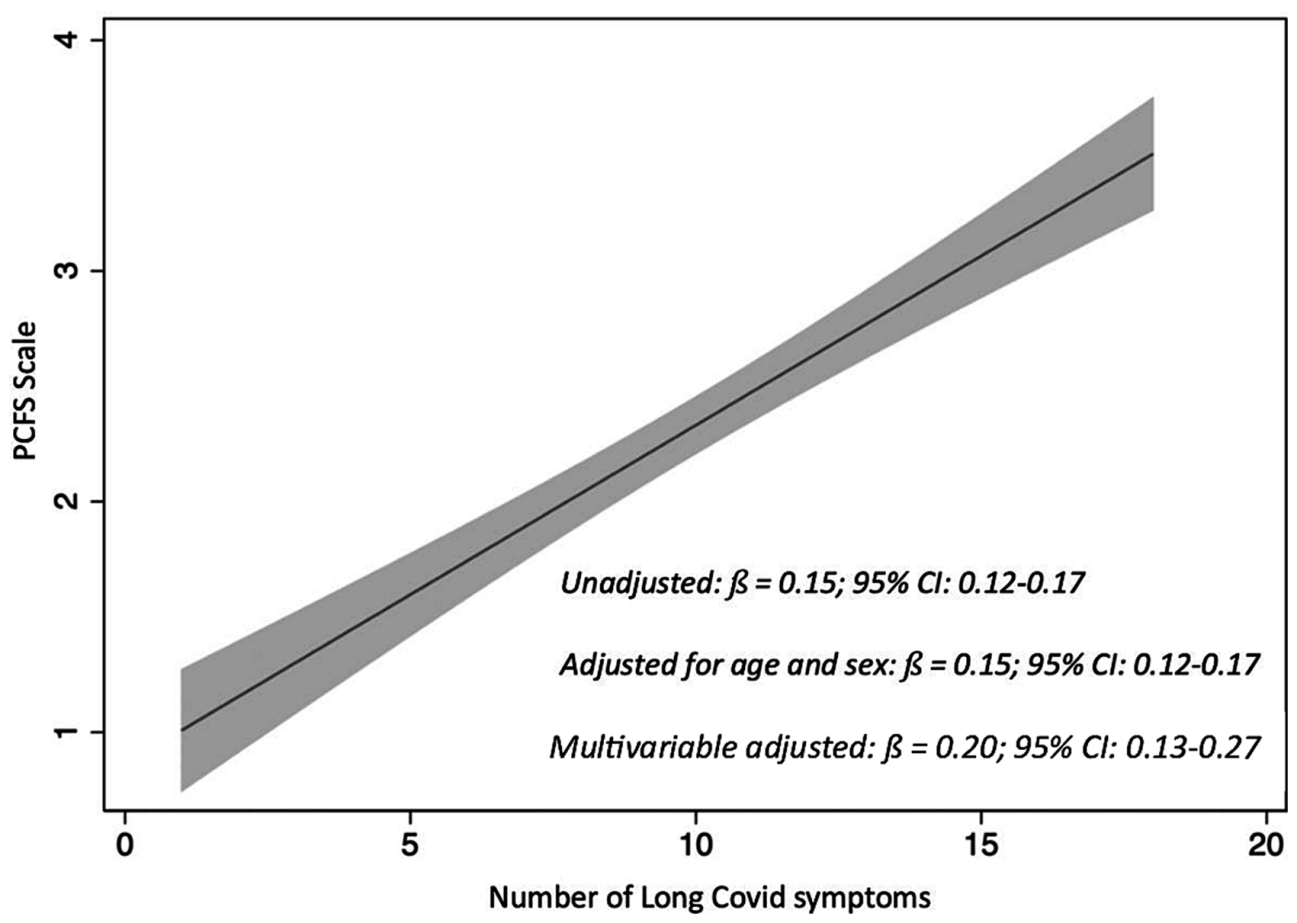
Association between number of Long COVID symptoms and Post-COVID-19 Functional Status (PCFS) scale. Estimates are from a fully adjusted linear regression controlling for age, sex, preexisting disease, BMI, smoking status, alcohol intake, physical activity, sedentary hours per day, sleep duration, MEDAS score, and vaccination status (influenza and COVID-19). The line shows the *β* per additional symptom, and the shaded areas show 95% CIs. N after imputation: 220.

In Figure 3, the graphical representation corresponds to the unadjusted (crude) association between the number of symptoms and the functional status scale (PCFS). The figure also shows the corresponding *β* coefficients and 95% confidence intervals from the unadjusted, age/sex-adjusted, and fully adjusted models. In the unadjusted model, we observed an association between the number of symptoms and functional status, with a *β* coefficient of 0.15 (95% CI, 0.12–0.17). This association remained stable after adjustment for age and sex (β = 0.15; 95% CI: 0.12–0.17) and persisted even in the fully adjusted multivariable model including sociodemographic, clinical, and lifestyle covariates (β = 0.17; 95% CI: 0.10–0.25).

Table 2 shows the linear regression analysis conducted to identify which symptoms were independently associated with greater functional limitation in individuals with LC.

**Table 2:** Contribution of individual symptoms to explained variability in daily functional limitations in Long COVID.

Symptom	Beta coefficient (unadjusted, 95% CI)	Beta coefficient (adjusted for the rest of the symptoms, 95% CI)	R^2^	Cumulative R^2^	F change
Fatigue	1.558 (1.215, 1.901)	0.705 (0.623, 0.788)	0.384	0.384	54.81
Dizziness	1.077 (0.813, 1.341)	0.545 (0.485, 0.605)	0.225	0.225	57.54
Memory loss	1.258 (0.970, 1.546)	0.482 (0.408, 0.556)	0.455	0.455	8.24
Depression	0.664 (0.377, 0.950)	0.327 (0.262, 0.392)	0.466	0.466	3.96
Other symptoms	0.721 (0.434, 1.008)	0.311 (0.070, 0.552)	0.513	0.513	6.49
Loss of smell/taste	−0.341 (−0.652, −0.031)	−0.332 (−0.384, −0.280)	0.479	0.479	5.22
Anxiety	0.611 (0.318, 0.904)	−0.213 (−0.279, −0.147)	0.455	0.455	0.27
Cough	0.287 (−0.037, 0.611)	−0.205 (−0.262, −0.148)	0.014	0.015	3.29
Dyspnea	0.803 (0.522, 1.084)	0.218 (0.155, 0.280)	0.398	0.398	4.96
Headache	0.973 (0.682, 1.264)	0.210 (0.144, 0.275)	0.415	0.415	5.98
Ophthalmological	0.840 (0.554, 1.125)	0.207 (0.149, 0.264)	0.497	0.497	2.17
Menstrual alterations	0.501 (0.187, 0.815)	0.247 (0.193, 0.301)	0.491	0.491	3.62
Brain fog	1.315 (0.990, 1.640)	0.141 (0.058, 0.224)	0.433	0.433	6.78
Myalgia	1.199 (0.894, 1.505)	0.126 (0.052, 0.200)	0.481	0.481	0.86
Sleep disorder	0.895 (0.588, 1.201)	−0.121 (−0.188, −0.054)	0.455	0.455	0.01
Palpitations	0.777 (0.491, 1.064)	−0.097 (−0.157, −0.036)	0.492	0.492	0.26
Gastrointestinal	0.812 (0.530, 1.093)	−0.059 (−0.120, 0.002)	0.479	0.479	0.00
Chest tightness	0.675 (0.380, 0.969)	0.044 (−0.018, 0.106)	0.482	0.482	0.10
Cardiovascular	0.556 (−0.076, 1.188)	0.016 (−0.098, 0.130)	0.492	0.492	0.02

Beta coefficients represent the magnitude and direction of the association between each symptom and the outcome. The 95% confidence intervals (CI) indicate the precision of each estimate. “Cumulative R^2^” reflects the proportion of explained variance in the dependent variable after each symptom is added to the model. “F change” corresponds to the increase in model fit with the inclusion of each new symptom block.

Symptoms were entered sequentially into the model to assess their incremental contribution to the explained variance in the outcome. In the first block, fatigue alone accounted for 38.4% of the variance in functional limitations (R^2^ = 0.384; *F* = 54.81; *p* < 0.001). The addition of dizziness increased the explained variance to 45.5% (*F* = 57.54; *p* < 0.001), and the inclusion of memory loss further increased it to 46.6% (*F* = 8.24; *p* < 0.001). Finally, the entry of a group of symptoms categorized as “other symptoms” further increased the total R^2^ to 51.3% (*F* = 6.49; *p* < 0.01). The last category includes less frequently reported manifestations that are not within the predefined domains of brain fog: neurological, digestive, ophthalmological, dermatological, and auditory symptoms; alopecia; memory impairment; or sleep disturbances.

In a sensitivity analysis excluding the “other symptoms” category, the explained variance slightly decreased to 49.7%. In addition, separate unadjusted linear regressions were conducted for each symptom to explore the potential multicollinearity among the predictors.

Symptoms that were not retained as independent predictors in the fully adjusted model—including dyspnea, headache, anxiety, depression, anosmia/dysgeusia, and sleep disturbances—did not show statistically significant associations once the main predictors (fatigue, dizziness, memory loss, and the composite “Other symptoms” group) were taken into account.

## 4 Discussion

This cross-sectional study examined functional limitations in relation to symptom count and symptom type among individuals with Long COVID. Our findings indicate that a higher number of persistent symptoms is associated with greater functional limitations, as measured using the *PCFS* scale. Moreover, the analysis revealed that fatigue, dizziness, and memory loss are the symptoms most strongly linked with this impairment.

In our study, the link between higher symptom count and worse functional status is consistent with the co-occurrence of multiple pathophysiological processes—such as chronic low-grade inflammation, immune dysregulation, autonomic imbalance, and neurocognitive impairment (22, 23)—which could reflect an accumulated burden on daily functioning. Previous research shows that these symptoms often coexist and may exacerbate one another, which may be accompanied by a lower quality of life and greater functional limitations (24–26).

In previous studies, fatigue has been associated with a lower quality of life in patients with systemic sclerosis (27), multiple sclerosis (28), and fibromyalgia (29). As a multidimensional symptom, it affects independence, social interaction, and occupational performance and is frequently reported alongside sleep disturbances, depressive symptoms, and anxiety, which complicate clinical management (30, 31).

Regarding dizziness—whose pathophysiology has been examined through vestibular and autonomic testing in patients with LC—peripheral and central vestibular dysfunction, neurological damage, dysautonomia, and cerebrovascular alterations have been described (32, 33). In other disorders, dizziness has been associated with poorer general and mental health, as well as occupational limitations and difficulties in daily activities (34). Similarly, studies involving patients with chronic and episodic vestibular disorders have reported a lower quality of life (35, 36).

Regarding memory loss, within the broader neurocognitive profile of LC (including dizziness and brain fog), difficulties in attention, processing speed, and executive function frequently co-occur, which may be accompanied by greater functional limitations (37, 38). Evidence shows that patients with cognitive impairment following COVID-19 often report difficulties in maintaining employment, managing daily activities, and sustaining interpersonal relationships, resulting in a reduction in quality of life (37, 39).

These findings suggest that symptoms such as fatigue, dizziness, and memory loss—although not always the most frequently reported—are most strongly linked to worse long-term functional limitations. In contrast, more common symptoms of LC such as dyspnea, headache, and sleep disturbances did not show independent associations with functional limitations, suggesting a more limited contribution to disability despite their clinical relevance.

Another particularly relevant aspect is the association between the number of self-reported symptoms and functional impairment as measured by the PCFS scale, which remained significant even after adjustment for sociodemographic, clinical, and lifestyle variables. Exploratory unadjusted analyses showed higher coefficients for individual symptoms, suggesting that the loss of significance in the multivariable model may reflect multicollinearity among predictors.

This finding suggests the potential usefulness of symptom count as a pragmatic indicator of functional limitations associated with LC, particularly in settings such as primary care or occupational health, where standardized functional assessments may not be readily available. These results are consistent with those reported by Schmidbauer et al. (40). However, whereas that study focused on the *Fatigue Assessment Scale (FAS)* (41) in an adult outpatient population with mild infection, our approach incorporated a more heterogeneous sample and broader functional metric, thereby increasing the clinical applicability of the results. In addition, the use of hierarchical regression models allowed for the identification of symptoms most strongly linked to functional limitations, an aspect that has not been explored previously.

From a public health perspective, these observational patterns highlight the heterogeneity of Long COVID and the need for stratified care models. Individuals with a higher symptom burden—particularly those reporting fatigue and neurocognitive symptoms—may warrant prioritized assessment and rehabilitation planning. Such approaches may be more effective in reducing functional limitations and promoting recovery.

This clinically oriented, functionality-centered approach increases the applicability of our findings in contexts such as medical evaluations and work disability assessments related to Long COVID. It may also help contextualize functional status, provide objective tools for assessing the functional limitations of symptoms in daily life, and support clinical decision-making regarding sick leave, clinical follow-up, and rehabilitation needs.

### 4.1 Strengths and limitations

This study has several strengths. It is based on a well-defined clinical sample with confirmed SARS-CoV-2 infection and employs a structured assessment of persistent symptoms alongside a validated measure of functional limitations. Stratification by symptom tertile and the use of hierarchical regression models allowed for a detailed analysis of both cumulative and individual symptom effects.

Nonetheless, some limitations of this study must be acknowledged. First, the cross-sectional design precludes causal inferences between symptoms and functional impairments. Second, the use of self-reported symptoms may introduce recall bias, although a structured questionnaire helps mitigate this risk. Third, selection bias cannot be ruled out, as participants who agreed to follow-up may have differed from those who did not. Fourth, although multiple covariates were adjusted, residual confounding from unmeasured variables (e.g., mental health history or socioeconomic status) cannot be entirely excluded. Finally, the absence of sequencing-confirmed variant assignments limits variant-specific interpretations. Although the most likely variant was inferred from calendar times and periods of dominant circulation, this proxy is susceptible to ecological misclassification and has been used only for descriptive contexts. Studies with individual-level variant data are required to test this hypothesis directly.

In summary, this study highlights that the number of LC symptoms and specific symptoms—particularly fatigue, dizziness, and memory loss—are strongly associated with greater functional limitations in individuals with Long COVID. Identifying these key indicators can help clinicians better stratify risks, guide targeted interventions, and support occupational and disability assessments. As healthcare systems continue to address the long-term consequences of COVID-19, incorporating symptom-specific evaluations into routine care may improve patient outcomes and quality of life. Longitudinal studies are needed to confirm these associations over time and to explore the underlying mechanisms sustaining persistent symptoms.

## 5 Conclusion

In summary, our study highlights that the number of LC symptoms and specific symptoms—particularly fatigue, dizziness, and memory loss—are associated with functional limitations. These findings may help clinicians in patient risk stratification and inform occupational health assessments. Prospective studies are required to evaluate causality and make accurate predictions.

## Data Availability

The raw data supporting the conclusions of this article will be made available by the authors, without undue reservation.
